# A Rare Case of Pure Primary Large Cell Neuroendocrine Carcinoma of the Gallbladder

**DOI:** 10.1155/2022/6956046

**Published:** 2022-05-21

**Authors:** Rodney E. Shackelford, Ekin Ozluk, Jehan Abdulsattar, Terry C. Lairmore, Quyen Chu, Eric X. Wei

**Affiliations:** ^1^Department of Pathology, University of South Alabama Health, Mobile, Alabama 36617, USA; ^2^Department of Pathology & Translational Pathobiology, LSU Health Sciences Center Shreveport, Shreveport, Louisiana 71130, USA; ^3^Department of Surgery, Louisiana State University Health Shreveport, Louisiana 71130, USA

## Abstract

Primary large cell neuroendocrine carcinoma (LCNEC) of the gallbladder is a rare malignancy which is often associated with non-LCNEC histologic components. Histologically “pure” LCNECs of the gallbladder are exceedingly rare with only 15 cases reported in the medical literature. Clinically, LCNECs present with abdominal pain and jaundice and follow an aggressive course with patients surviving a median of 15 months following initial diagnosis. To our knowledge, we present the 16^th^ case of a histologically pure LCNEC in a 62-year-old African American male who was successfully treated surgically. After discharge, he was subsequently lost to follow-up. Due to the extreme rarity of such disease entity, accurate diagnosis and proper management are essential for the best clinical outcome.

## 1. Introduction

The current World Health Organization (WHO) classification of neuroendocrine tumors of the gallbladder includes grade I and II neuroendocrine tumors, small cell neuroendocrine carcinoma, large cell neuroendocrine carcinoma (LCNEC), mixed adenoneuroendocrine carcinoma, goblet cell carcinoid, and tubular carcinoid tumors [1]. Histologically, neuroendocrine tumors frequently grow in trabecular or organoid patterns, often with rosettes and with “salt and pepper” nuclear patterns. The more poorly differentiated and clinically aggressive subtypes show a high mitotic index. Neuroendocrine tumors typically show immunoreactivity towards the neuroendocrine markers chromogranin, synaptophysin, CD56, Leu 7, and neuron-specific enolase [1–5]. LCNECs are composed of large cells that are usually two to three times larger than those of small cell neuroendocrine tumors and show brisk mitotic activity, with hyperchromatic nuclei and prominent nucleoli [1–5].

Primary neuroendocrine tumors of the gallbladder are rare, totaling only 0.5% of all neuroendocrine tumors and 2.1% of all gallbladder malignancies [2]. LCNECs were first described in 1991 when pulmonary cases were reported [3]. LCNECs of the gallbladder are extremely rare and are commonly composed of neuroendocrine cells with other associated histologies, including adenocarcinoma, adenosquamous carcinoma, and mucinous adenocarcinoma components [4]. LCNECs composed solely of enteroendocrine features (“pure” LCNECs) are exceptionally rare, with only 15 cases described in the medical literature [5]. Here, to our knowledge, we describe the 16^th^ case of a primary gallbladder LCNEC composed solely of large neuroendocrine carcinoma cells.

## 2. Case Presentation

**Figure 1:**
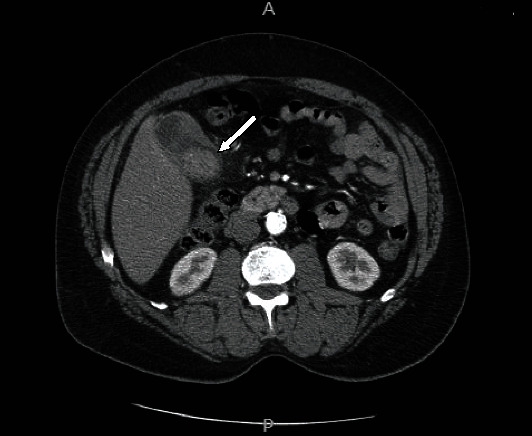
A noncontrast CT scan of the patient's abdomen revealing thickening of the gallbladder wall with a possible polyp (white arrow).

**Figure 2:**
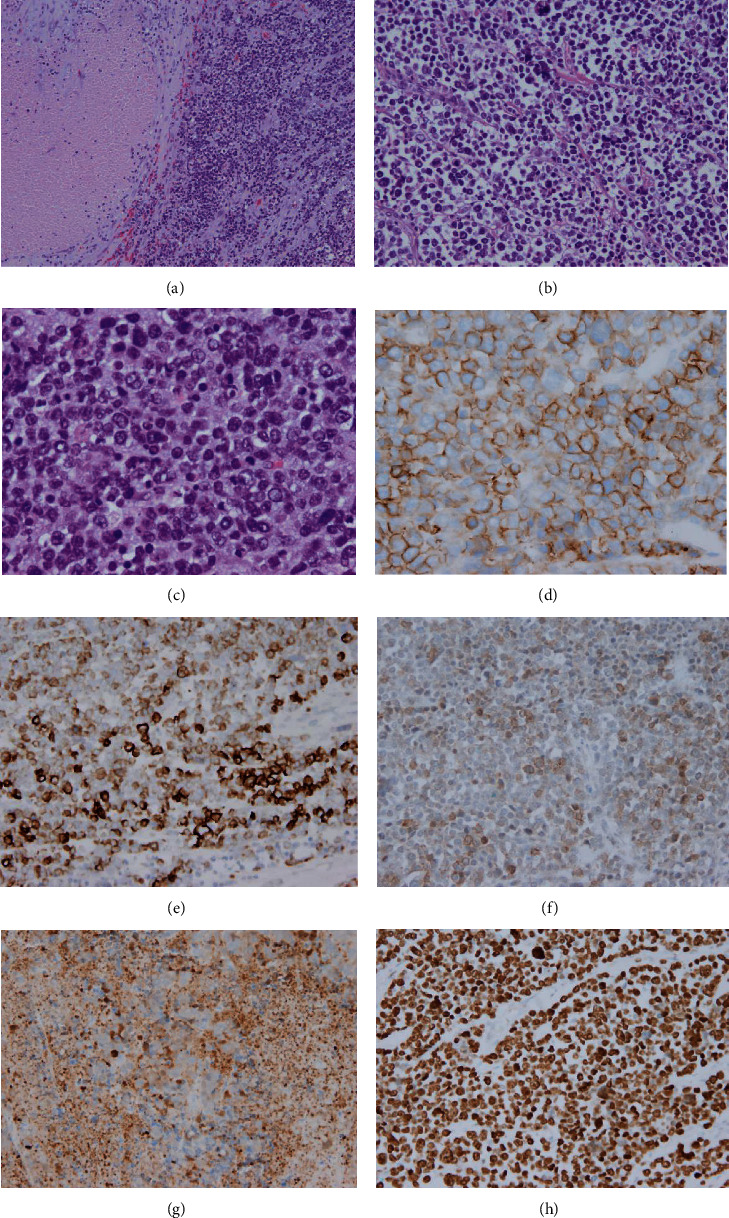
Representative H&E sections of the gallbladder LCNEC and some of the immunostains performed. Very low-power H&E view (20x) of the LCNEC showing necrosis and tumor cells (a), low-power H&E section (40x) showing the trabecular growth pattern (b), and a middle-power view (200x) showing the neuroendocrine features of the LCNEC (c). Sections (d–h) show the CD56, AE1/AE3, c-kit, neuron-specific enolase, and Ki-67 immunostains, respectively.

A 62-year-old African American male presented with a history of acute vomiting and right upper-quadrant and epigastric pain, one day before admission to the hospital. His pain had become so unbearable that he was prompted to call an ambulance. The patient's previous medical history included hypertension, hyperlipidemia, atherosclerosis, and type II diabetes, all of which were medically managed. He also had an extensive 40-year smoking history. The patient did not reveal any significant family medical history. His physical exam was significant for high blood pressure of 190/96 mmHg and guarding on palpation of the abdomen. His serum chromogranin or other neuroendocrine markers were not tested. Initial imaging studies were performed with ultrasound of the abdomen, which was significant for hepatomegaly and cholelithiasis, without evidence of cholecystitis. A noncontrast abdominal CT scan was performed where stones, a possible gallbladder polyp, and thickening of the gallbladder wall were identified, which were concerning for malignancy (Figure 1). A laparoscopic cholecystectomy was performed, and the gallbladder was found to be grossly distended, partially filled with multiple multifaceted calculi, containing a central polypoid mass roughly 3.0 cm in size and exhibiting focal thickening of the gallbladder wall. An attached gallbladder lymph node partially involved by metastatic tumor cells was also identified. Histologically, the tumor showed extensive necrosis and large cells with salt and pepper nuclei, prominent nucleoli, and a trabecular growth pattern (Figures 2(a)–2(c)). Immunohistochemical staining for AE1/AE3, chromogranin, synaptophysin, CD20, CD56, DOG-1, Ki-67, MART-1, S-100, SOX-10, TTF-1, neuron-specific enolase (NSE), and c-kit were performed on the polypoid mass. The tumor cells were immunoreactive to CD56 (membranous), AE1/AE3, c-kit, and NSE (cytoplasmic) (Figures 2(d)–2(g)) and negative for CD20, chromogranin, synaptophysin, DOG-1, S-100, and other markers performed, consistent with a LCNEC [1–5]. The Ki-67 immunostaining revealed a brisk miotic index with over 95% immunopositivity (Figure 2(h)). The tumor was staged as pT3, pN1. After the sixth day of hospitalization postoperatively, the patient recovered without further complications and was sent home with scheduled follow-up. His medications included treatment regimens for his hypertension, hyperlipidemia, and diabetes without further chemotherapy. After the discharge, the patient was subsequently lost to follow-up.

## 3. Discussion

Here, we presented a 62-year-old African American male with pure LCNEC of the gallbladder. The histological features and positive immunostainings for AE1/AE3, CD56, c-kit, and NSE and high proliferation index at 95% would support the diagnosis of LCNEC. Negative synaptophysin and chromogranin expression is unusual and may suggest poor differentiation of the tumor. Negative S-100 expression excludes a high-grade peripheral nerve sheath tumor. Although the tumor cells express c-kit, negative DOG-1 staining rules out an epithelioid gastrointestinal stomal tumor (GIST). The expression of c-kit in tumor cells may indicate tumor origin from undifferentiated stem cells. The fact that the tumor cells are negative for CD20 also removes the consideration of a large B cell lymphoma. Neuroendocrine tumors of the gallbladder are rare with LCNEC tumors of this organ being very uncommon [1, 5]. “Pure” gallbladder LCNECs containing only the large cell neuroendocrine histology are exceedingly rare, with only 15 cases recorded in the medical literature [5]. Neuroendocrine cells are not normally found in the gallbladder, which is a likely reason why these tumors are so rare in this organ [6–8]. How these tumors arise in the gallbladder is unknown; however, it has been hypothesized that these malignancies may arise from undifferentiated stem cells or from chronic inflammation such as cholecystitis leading to intestinal or gastric metaplasia and neuroendocrine cell formation with later malignant transformation [5–7]. LCNECs have presented in patients between the ages of 55 to 76 years, usually with symptoms similar to the more common gallbladder malignancies, including jaundice and abdominal pain/discomfort [5]. Common radiological findings include a mass in place of the gallbladder, focal or diffuse gallbladder wall thickening, and direct invasion of the tumor into the liver or metastasis to the surrounding lymph nodes [1]. LCNECs are typically very aggressive, with a median survival time of 15 months and with a range of 21 days to 69 months [5]. For qualifying patients, optimal treatment for these tumors is complete surgical resection with surrounding lymph node clearance and hepatic lobectomy, with recurrences being very common [5]. In the event of an unresectable tumor, systemic chemotherapy is the next treatment option, which employs the same platinum-based chemotherapy regimen, often with radiation therapy, as is used for small cell neuroendocrine carcinoma treatment [5, 8]. The efficacy of traditional chemotherapy with additional radiotherapy remains unclear due to insensitivity of general neuroendocrine tumors to radiotherapy; however, this is a treatment option for patients with multiple metastases or an unresectable tumor [9]. Recently, immunotherapy with ipilimumab and nivolumab has shown some promise in a small phase II clinical trial treating LCNECs [10]. Due to the rarity of these tumors, especially of pure LCNECs of the gallbladder, there is much that is unknown about how these extremely rare tumors should be treated [8, 10]. An accurate diagnosis of LCNEC is also essential as the treatment for these tumors is different than that used for the more common adenocarcinomas of the gallbladder [5, 8, 11]. In conclusion, histologically pure LCNEC tumors of the gallbladder are exceedingly rare [5, 11]. The unique nature of this tumor requires that further studies be utilized to optimize its treatment. In particular, immune and DNA sequence analyses, multimodal treatment regimes, and immune therapies will likely play an important role in the future treatment of these rare malignancies [5, 11].

## References

[1] Eltawil K. M., Gustafsson B. I., Kidd M., Modlin I. M. (2010). Neuroendocrine tumors of the gallbladder. *Journal of Clinical Gastroenterology*.

[2] Papotti M., Cassoni P., Sapino A., Passarino G., Krueger J. E., Albores-Saavedra J. (2000). Large cell neuroendocrine carcinoma of the gallbladder: report of two cases. *The American Journal of Surgical Pathology*.

[3] Travis W. D., Linnoila R. I., Tsokos M. G. (1991). Neuroendocrine tumors of the lung with proposed criteria for large-cell neuroendocrine carcinoma. An ultrastructural, immunohistochemical, and flow cytometric study of 35 cases. *The American Journal of Surgical Pathology*.

[4] Liu W., Wang L., He X. D., Feng C., Chang X. Y., Lu Z. H. (2015). Mixed large cell neuroendocrine carcinoma and adenocarcinoma of the gallbladder: a case report and brief review of the literature. *World Journal of Surgical Oncology*.

[5] Tidjane A., Boudjenan N., Bengueddach A. (2021). Pure large cell neuroendocrine carcinoma of the gallbladder, is surgical relentlessness beneficial? A case report and literature review. *International Cancer Conference Journal*.

[6] Murakami M., Katayama K., Kato S. (2016). Large-cell neuroendocrine carcinoma of the common bile duct: a case report and a review of literature. *Surgical Case Reports*.

[7] Park S. B., Moon S. B., Ryu Y. J. (2014). Primary large cell neuroendocrine carcinoma in the common bile duct: first Asian case report. *World Journal of Gastroenterology*.

[8] Iyoda A., Makino T., Koezuka S., Otsuka H., Hata Y. (2014). Treatment options for patients with large cell neuroendocrine carcinoma of the lung. *General Thoracic and Cardiovascular Surgery*.

[9] Modlin I. M., Kidd M., Drozdov I., Siddique Z. L., Gustafsson B. I. (2008). Pharmacotherapy of neuroendocrine cancers. *Expert Opinion Pharmacotherapy*.

[10] Patel S. (2019). A phase II basket trial of nivolumab and ipilimumab in rare tumors (NET cohort). *American Association of Cancer Research*.

[11] Buscemi S., Orlando E., Damiano G. (2016). “Pure” large cell neuroendocrine carcinoma of the gallbladder. Report of a case and review of the literature. *International Journal of Surgery*.

